# Back to the Roots: Safety and Tolerability of Standardised Ashwagandha (*Withania somnifera*) Root Extract in Healthy Adults—A Systematic Review of Biomarkers and Adverse Events

**DOI:** 10.3390/ph19050725

**Published:** 2026-05-02

**Authors:** Olivia C. Coope, Mark E. T. Willems, Alex Levington, Mark J. Tallon, Blanca Roman-Viñas, Tilly J. Spurr

**Affiliations:** 1 Blanquerna School of Health Sciences, Ramon Llull University, 08025 Barcelona, Spain; 2 School of Sport, Science and Engineering, University of Chichester, College Lane, Chichester PO19 6PE, UK; m.willems@chi.ac.uk (M.E.T.W.); m.spurr@chi.ac.uk (T.J.S.); 3 Strength and Nutrition Ltd., Brighton BN3 8PS, UK; 4 Legal Products Group Ltd., Ely CB7 4EX, UK; drtallon@legalfoods.com; 5 Department of Physical Activity and Sport Sciences, Psychology, Education and Sport Sciences, Blanquerna, Universitat Ramon Llull, 34 Cister, 08022 Barcelona, Spain; blancarv@blanquerna.url.edu

**Keywords:** ashwagandha, standardised root extract, safety, tolerability, biomarkers, adverse events, herbal supplements, clinical trials

## Abstract

**Background:** Standardised Ashwagandha root extract (SARE), characterised by its content of bioactive withanolides, is widely used for its antioxidant and adaptogenic properties; however, recent case reports have raised safety concerns, primarily involving non-standardised or multi-ingredient formulations. This systematic review evaluated the safety and tolerability of SARE in healthy adults, with a focus on clinical biomarkers and adverse event reporting. **Methods:** Randomised trials were identified through searches of PubMed, Web of Science and Google Scholar, published from 2010 to April 2026. Studies administering single-ingredient, standardised root-only extracts to generally healthy populations were included. Risk of bias was assessed using the Cochrane RoB 2 tool. **Results:** Twenty-three studies with a total of 2317 participants met the inclusion criteria, with doses ranging from 125 to 600 mg/day and intervention durations from a single dose to 180 days. Across studies, hepatic, renal, haematological, endocrine, and cardiovascular biomarkers remained within normal clinical ranges, with no clinically meaningful adverse alterations reported. Reductions in cortisol were consistently observed, while increases in testosterone remained within physiological ranges. No serious adverse events attributable to SARE were reported. Mild adverse events, including gastrointestinal discomfort, headache, and transient drowsiness, were infrequently reported and occurred in both intervention and comparator groups. **Conclusions:** SARE was well tolerated in healthy adults at the studied doses and durations. However, limited long-term data (>180 days) and heterogeneity in study design and reporting warrant further large-scale, standardised trials to confirm safety across extended use and diverse populations. The review is registered in the PROSPERO database with ID CRD420261337116.

## 1. Introduction

The use of herbal supplements has increased substantially over the past two decades, both in the general population and within physically active and health-conscious individuals [1]. This trend is reflected in the rapid expansion of the global dietary supplement market, which was valued at approximately USD 209.5 billion in 2025 and is projected to reach USD 393.6 billion by 2033 [2]. They are commonly consumed to support stress management, sleep, recovery, hormonal balance and overall well-being [3]. However, unlike pharmaceutical agents, many botanical supplements have widespread use with limited evaluation of their safety, tolerability and biological effects in humans [4]. This gap is relevant for supplements that exert physiological effects across multiple systems, where subtle alterations in biomarkers may have important implications for long-term health [5,6].

Ashwagandha (*Withania somnifera*) is a plant-derived source of bioactive compounds widely used as an herbal supplement, with a long history of traditional use in Ayurvedic medicine, a traditional Indian system of plant-based and holistic medicine [7,8]. Traditionally, Ashwagandha has been used for its powerful antioxidant [9] and adaptogenic properties, with the root being the most commonly utilised and studied component [10]. Ashwagandha supplements are typically standardised according to their withanolide content, a group of steroidal lactones considered responsible for much of the plant’s biological activity [11]. More than 40 withanolides and related glycowithanolides have been identified in *Withania somnifera*, including withaferin A, withanolide A and withanoside IV, which exhibit diverse pharmacological properties including anti-inflammatory, neuroprotective and endocrine-modulating effects [12]. Growing interest in Ashwagandha has led to an expanding body of human trials investigating its effects on stress, sleep, cognitive function, physical performance, hormonal regulation and recovery [13]. These effects are thought to be mediated primarily through modulation of the hypothalamic–pituitary–adrenal axis, resulting in reductions in cortisol and improved stress resilience [14]. Additional proposed mechanisms include antioxidant and anti-inflammatory actions mediated through modulation of nuclear factor-κB signalling, regulation of sex hormone balance and improvements in mitochondrial function and cellular energy metabolism [15].

Despite the increasing popularity of Ashwagandha root extract supplementation and recent research showing biological safety after 12 months of supplementation [16], concerns regarding its safety remain, driven by isolated case reports of hepatotoxicity [17]. Human trials typically administer doses ranging from 300 to 600 mg per day of standardised root extract containing approximately 5% withanolides, although considerable variability exists between commercial formulations in terms of extraction methods, plant part used and phytochemical composition. Currently, various reports involve non-standardised products, multi-ingredient formulations, or preparations containing leaf material rather than root extract [18]. This distinction is critical, as the phytochemical composition and biological effects of Ashwagandha leaf and root differ substantially: a recent in vitro study using primary human hepatocytes demonstrated that ethanolic Ashwagandha leaf extract induced dose- and time-dependent hepatocellular toxicity, suggesting a potential risk to liver health at higher concentrations. In contrast, the root extract did not exhibit direct hepatotoxic effects but was shown to modulate cytochrome P450 3A4 (CYP3A4) activity, an enzyme responsible for the metabolism of approximately 30–50% of clinically used drugs, indicating a possible mechanism for drug-herb interactions rather than intrinsic toxicity [19]. Standardised root-only extracts, particularly those with defined withanolide concentrations, are the formulations most commonly investigated in controlled clinical trials [20] and are therefore the most relevant for evidence-based safety assessment.

Many clinical studies examining Ashwagandha supplementation incorporate routine clinical safety assessments, including biomarkers such as liver enzymes (e.g., alanine aminotransferase [ALT] and aspartate aminotransferase [AST], indicators of hepatic integrity), renal function indices (e.g., serum creatinine and blood urea nitrogen, markers of kidney filtration), haematological parameters (e.g., haemoglobin and leukocyte counts, reflecting blood and immune status) and hormonal markers (e.g., cortisol or thyroid hormones), which help detect potential physiological disturbances associated with supplementation [21].

The aim of this systematic review is to critically evaluate and synthesise evidence on the safety and tolerability of standardised Ashwagandha root extract (SARE) in healthy adult populations, with a particular focus on clinical biomarkers and adverse event reporting. This review aims to inform practitioners and regulatory stakeholders and to support evidence-based recommendations regarding the use of Ashwagandha in healthy individuals. This systematic review is registered in the PROSPERO database with ID number CRD420261337116.

## 2. Materials and Methods

### 2.1. PICOS

This systematic review adhered to the Preferred Reporting Items for Systematic Reviews and Meta-Analyses (PRISMA) 2020 statement [22]. A completed PRISMA checklist is provided in Supplementary Materials S1. The review protocol was registered with PROSPERO. The eligibility criteria for this systematic review were defined *a priori* using the Population, Intervention, Comparator, Outcomes and Study design (PICOS) framework to maintain a structured and transparent study selection process [23]. The review focused on adult populations considered generally healthy, without a diagnosed disease, to evaluate the safety and tolerability of SARE under conditions representative of typical supplementation use. Eligible interventions were restricted to SARE administered as a single-ingredient product, irrespective of dose, formulation, or duration, to minimise confounding from multi-ingredient supplements or concurrent pharmacological agents. The comparators included placebo, sham interventions, or no-intervention control conditions, allowing for the assessment of SARE-specific effects. The outcomes of interest centred on biomarkers including hepatic, renal, haematological and hormonal parameters, safety and tolerability, with a focus on adverse event reporting. Eligible studies were randomised, parallel-group, placebo-controlled, or active-controlled trials. Single-arm studies, case reports, case series, and non-randomised designs were excluded to ensure robust causal inference regarding safety outcomes. No minimum intervention duration was specified to capture both acute and chronic safety profiles. Studies ranged from single-dose pharmacokinetic investigations to extended supplementation trials.

For the purpose of this review, “healthy” participants were defined as adults without a diagnosed active disease or acute pathological conditions. Populations experiencing non-pathological or life-stage-related conditions, such as perceived psychological stress, mild cognitive impairment, menopausal status, or age-related frailty, were considered eligible, as these states do not constitute active diseases and are commonly examined in preventive or wellness-focused clinical research. However, the inclusion of populations with conditions such as these may introduce clinical heterogeneity and should be considered when interpreting safety outcomes. The specific PICOS criteria applied in this review are detailed below.

P—Population

Healthy adults of any sex, aged ≥18 years, were considered healthy with no active disease.

I—Intervention

Administration of SARE alone, in any dose, form, or duration, as a single-ingredient intervention (i.e., not in combination with other herbal products, supplements, or pharmacological agents).

C—Comparator

A comparison between an experimental group receiving SARE and a control group, including:

- Placebo
- Sham intervention
- No intervention

O—Outcomes

Outcomes related to safety and tolerability, such as the following, were evaluated:

- Adverse events (frequency, type and severity)
- Clinical safety biomarkers from human serum, urine or saliva samples

S—Study design

Randomised controlled trials (RCTs), including blinded and open-label randomised designs (e.g., crossover or parallel-group), were eligible for inclusion.

### 2.2. Search Keywords

A systematic keyword-based search strategy was developed to identify all relevant clinical trials examining the safety and tolerability of SARE in healthy adult populations. Search terms were constructed using controlled vocabulary and free-text keywords related to the intervention (*Withania somnifera*/Ashwagandha), extract standardisation, safety outcomes and clinical study design. Boolean operators were applied to combine terms consistently across databases, and search strategies were adapted to the specific indexing requirements of each platform. Scopus was not included in the search strategy due to overlap in journal indexing with Web of Science and PubMed. The combination of PubMed and Web of Science has broad coverage of the literature, while Google Scholar was used to enhance sensitivity and identify additional records. The complete database-specific search strings used in this review are presented in Table 1.

### 2.3. Data Extraction

Data were extracted between October 2025 and April 2026 using a structured and standardised approach to ensure consistency across studies. The extracted information included the author and year of publication, country, study design, participant characteristics, sample size, details of the intervention and comparator, duration of supplementation, outcomes assessed, safety and tolerability measures, adverse events, and main findings. Studies published between 2010 and April 2026 were considered for inclusion.

Data extraction was independently performed by two reviewers (O.C.C. and A.L.) using a standardised, piloted extraction form. The extracted data included study characteristics (design, setting, and duration), participant demographics, intervention details (dose, withanolide content, and manufacturer), outcome measures (biomarkers and adverse events), and results. Discrepancies were resolved through discussion between the two reviewers. When data were unclear or missing, the corresponding authors were contacted via email with a two-week response window.

### 2.4. Outcomes

The primary outcomes of interest in this systematic review were measures of biomarkers and safety associated with SARE supplementation. These included changes in clinical safety biomarkers, such as liver enzymes, renal function markers, haematological indices, inflammatory markers, and hormonal parameters, as well as the frequency, type, and severity of adverse events. Secondary outcomes related to physiological, cognitive, and performance-related measures are described where relevant to contextualise safety findings.

Clinically significant changes in biomarkers were defined *a priori* as follows, according to the Common Terminology Criteria for Adverse Events (CTCAE):

- Hepatotoxicity: ALT or AST > 3× upper limit of normal (ULN), or meeting Hy’s Law criteria (ALT > 3× ULN with total bilirubin > 2× ULN).
- Renal impairment: Serum creatinine increase ≥ 0.3 mg/dL from baseline or ≥1.5× baseline value.
- Thyroid dysfunction: TSH outside reference range (typically 0.4–4.0 mIU/L) with corresponding free T4 abnormalities.
- Haematological abnormalities: Values outside laboratory reference ranges with clinical significance (e.g., anaemia: haemoglobin < 12 g/dL in women, <13 g/dL in men).

The adverse events were classified according to severity (mild, moderate, or severe) and relationship to the intervention (definitely, probably, possibly, unlikely, or unrelated) as reported by the study authors.

### 2.5. Study Selection

A total of 702 records were identified through database searching, including PubMed (*n* = 51), Google Scholar (*n* = 618) and Web of Science (*n* = 33). After removing duplicate records (*n* = 145), 557 studies remained for title and abstract screening. Of these, 514 records were excluded because they did not meet the inclusion criteria. The high proportion of records retrieved from Google Scholar reflects its broad indexing; however, this may reduce reproducibility and increase the risk of selection bias compared with structured databases.

**Figure 1:**
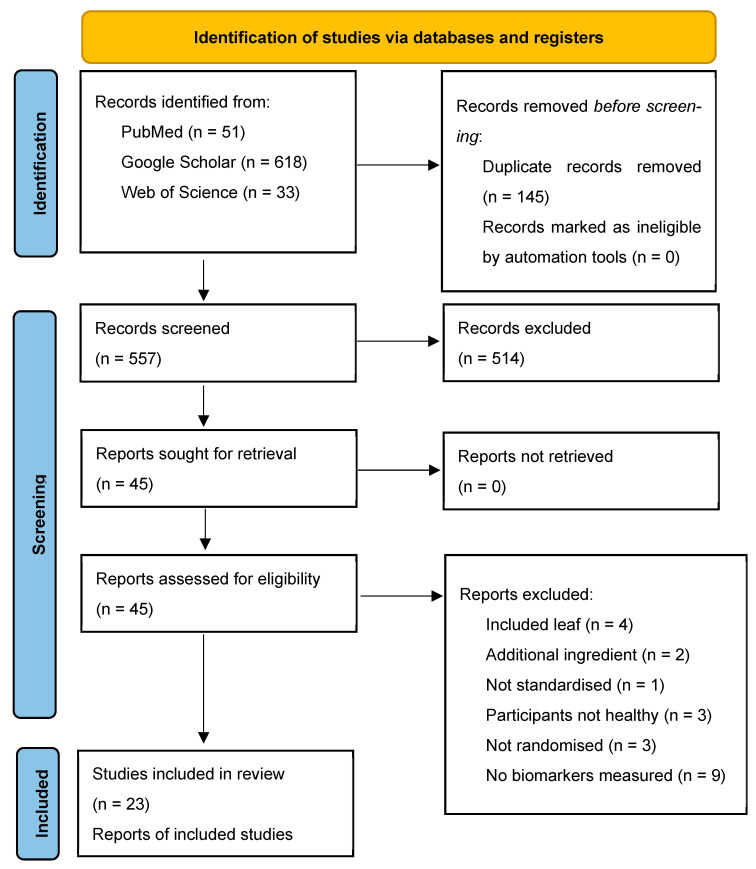
PRISMA 2020 flow diagram of study identification, screening, eligibility, and inclusion of clinical trials assessing SARE safety in healthy adults.

The full texts of 45 reports were sought and subsequently assessed for eligibility. Twenty-two reports were excluded for the following reasons: inclusion of leaf rather than root extract (*n* = 4), presence of additional active ingredients (*n* = 2), lack of standardisation (*n* = 1), inclusion of non-healthy participants (*n* = 3), non-randomised trials (*n* = 3) or absence of biomarker measurements from saliva, urine or serum (*n* = 9). In total, 23 studies met the eligibility criteria and were included in this review [24,25,26,27,28,29,30,31,32,33,34,35,36,37,38,39,40,41,42,43,44,45,46]. The study selection process is summarised in Figure 1.

### 2.6. Certainty of Evidence

The certainty of evidence for each outcome was assessed using the Grading of Recommendations, Assessment, Development and Evaluation (GRADE) framework. Evidence derived was initially rated as high certainty and subsequently downgraded based on five domains: risk of bias, inconsistency, indirectness, imprecision, and publication bias. Certainty was classified as high, moderate, low, or very low. Given the absence of meta-analysis, assessments were based on qualitative synthesis of effect direction, consistency, and study characteristics.

### 2.7. Statistical Analysis

Due to heterogeneity in study design, intervention protocols and outcome reporting, a meta-analysis was not conducted. A structured qualitative synthesis was performed. Data on biomarker changes and adverse events were extracted and summarised descriptively. Where reported, between-group differences, percentage changes, and statistical outputs were presented without reanalysis. Findings were synthesised by biomarker category, and adverse events were summarised using reported frequencies, noting variability in reporting across studies. Statistical significance was interpreted according to the thresholds defined within individual studies (typically *p* < 0.05).

## 3. Results

### 3.1. Study Characteristics

**Figure 2:**
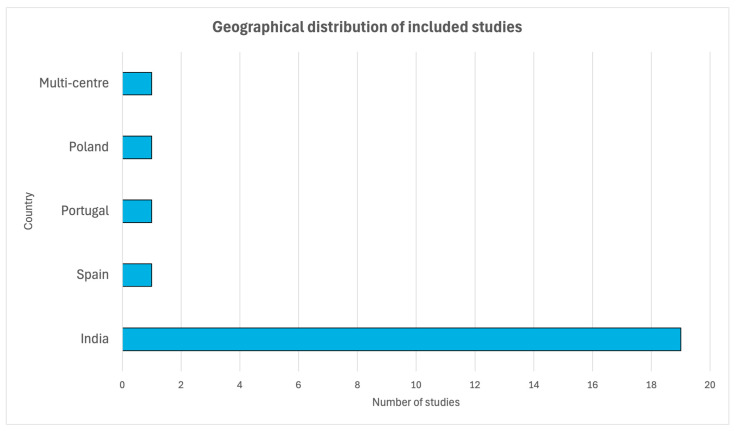
Geographical distribution of included studies.

The 23 studies selected for this review were published between 2012 and 2026 and were conducted primarily in India (*n* = 19), with additional studies conducted in Spain (*n* = 1), Poland (*n* = 1), (*n* = 1), and one multi-centre multinational trial conducted across India, the United States, Africa, Portugal, and Australia (*n* = 1). Only studies published in English were included. Grey literature (e.g., conference abstracts, preprints, and unpublished data) was not systematically searched. The predominant study design was the randomised controlled trial (RCT; *n* = 21), one open-label randomised crossover (single-dose) study, and one open-label study. The included studies investigated a range of populations, including healthy adults experiencing chronic stress, athletic and physically active adults, elderly individuals with frailty, menopausal women, male and female athletes, and adults with insomnia, sexual dysfunction, or stress and anxiety; none of the participants had an active disease. Participants with no evidence of active disease were considered healthy, consistent with the definition of health as the absence of disease or impairment [47]. The participants were functionally healthy but with non-pathological conditions. Of the included trials, most studies included mixed-sex cohorts, with several conducted exclusively in male participants and two in female-only populations. A description of the study characteristics is provided in Table 2, and the geographical distribution of included studies is illustrated in Figure 2.

SARE dosing protocols varied across studies, ranging from a single-dose administration of 600 mg to chronic supplementation regimens of 125–600 mg/day. Intervention durations ranged from a single dose to 180 days, with the majority of trials administering 600 mg/day over periods of 6–12 weeks. Collectively, the included studies comprised a total of 2317 participants, with individual sample sizes ranging from 14 to 1002 participants. SARE supplementation was well tolerated. Mild adverse events were reported in several studies, most commonly gastrointestinal discomfort, headache, mild upper respiratory symptoms, or “transient drowsiness”.

Sixteen studies utilised KSM-66, a root-only aqueous extract standardised to ≥5% withanolides, administered predominantly at a dose of 600 mg/day [24,25,26,27,30,31,32,33,35,37,39,40,42,43,44,46]. The remaining studies utilised alternative SARE formulations, including sustained-release preparations [28,29], proprietary extracts such as Zenroot™ standardised to 1.5% withanolides [38], ASVAMAN^®^ standardised to 2.5% withanolides [41], and Agewel™ standardised to 1.5% withanolides [36], as well as LongeFera™ standardised to ≥2.5% withanolides [34]. One further trial [45] used a standardised Ashwagandha root extract at 600 mg/day but did not specify a proprietary formulation. The pharmacokinetic study [28] compared a sustained-release formulation with a matched-dose KSM-66 comparator under single-dose conditions.

A broad range of physiological, biochemical, and performance-related biomarkers was assessed, including endocrine, inflammatory, haematological, hepatic, renal, and cardiorespiratory outcomes. Placebo-controlled trials generally employed matched oral formulations. No serious adverse events attributable to SARE were reported, and no clinically meaningful changes in safety-related biomarkers or vital signs were observed, although subtle or longer-term effects cannot be excluded. Mild adverse events occurred in both intervention and comparator groups. Details of assessed biomarkers and adverse events are presented in Table 3.

### 3.2. Risk of Bias Assessment

**Figure 3:**
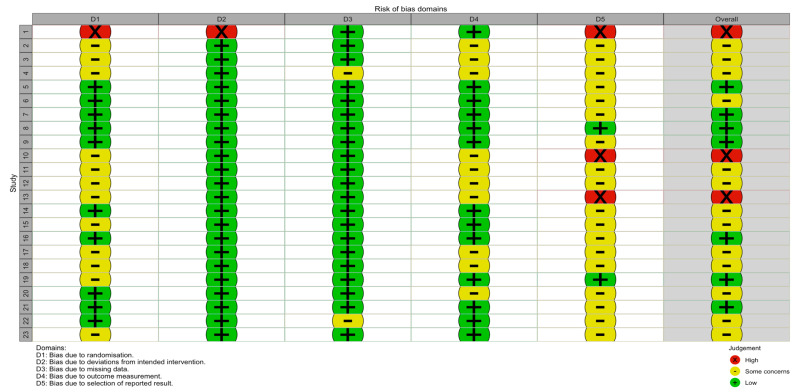
Risk of bias assessment (Cochrane RoB 2 tool) evaluating SARE safety outcomes. Studies were numbered sequentially by author and year of publication. Study 1 corresponded to Alluri et al. (2021) [28], study 2 to Chandrasekhar et al. (2012) [24], study 3 to Chauhan et al. (2022) [33], study 4 to Choudhary et al. (2015) [26], study 5 to Coope et al. (2026) [43], study 6 to Ferreira et al. (2026) [45], study 7 to Gopukumar et al. (2021) [29], study 8 to Jówko et al. (2025) [42], study 9 to Langade et al. (2021) [30], study 10 to Mahadevan et al. (2025) [38], study 11 to Mutha et al. (2025a) [40], study 12 to Mutha et al. (2025b) [39], study 13 to Naik et al. (2024) [35], study 14 to Pakhale et al. (2026) [46], study 15 to Puttaswamy et al. (2025) [41], study 16 to Raut et al. (2024) [36], study 17 to Salve et al. (2019) [27], study 18 to Tiwari et al. (2021) [31], study 19 to Vaidya et al. (2025) [34], study 20 to Vani et al. (2026) [44], study 21 to Verma et al. (2021) [32], study 22 to Verma et al. (2024) [37] and study 23 to Wankhede et al. (2015) [25].

Risk of bias was assessed for all 23 included studies using the Cochrane Risk of Bias 2 (RoB 2) tool across five domains: bias arising from the randomisation process (D1), bias due to deviations from intended interventions (D2), bias due to missing outcome data (D3), bias in the measurement of the outcome (D4) and bias in selection of the reported result (D5) [48]. Overall, most studies were judged to be at low risk of bias or to raise some concerns, with a small number assessed as being at high risk of bias. Detailed RoB 2 judgments are presented in Figure 3. Studies are numbered sequentially according to the author and year of publication. Study 1 corresponds to Alluri et al. (2021) [28], study 2 to Chandrasekhar et al. (2012) [24], study 3 to Chauhan et al. (2022) [33], study 4 to Choudhary et al. (2015) [26], study 5 to Coope et al. (2026) [43], study 6 to Ferreira et al. (2026) [45], study 7 to Gopukumar et al. (2021) [29], study 8 to Jówko et al. (2025) [42], study 9 to Langade et al. (2021) [30], study 10 to Mahadevan et al. (2025) [38], study 11 to Mutha et al. (2025a) [40], study 12 to Mutha et al. (2025b) [39], study 13 to Naik et al. (2024) [35], study 14 to Pakhale et al. (2026) [46], study 15 to Puttaswamy et al. (2025) [41], study 16 to Raut et al. (2024) [36], study 17 to Salve et al. (2019) [27], study 18 to Tiwari et al. (2021) [31], study 19 to Vaidya et al. (2025) [34], study 20 to Vani et al. (2026) [44], study 21 to Verma et al. (2021) [32], study 22 to Verma et al. (2024) [37], and study 23 to Wankhede et al. (2015) [25].

Bias arising from the randomisation process (D1) was the most frequent source of concern. Several studies were rated as having some concerns due to insufficient reporting of random sequence generation or allocation concealment, and a high risk of bias was identified in Study 1. Bias due to deviations from intended interventions (D2) was predominantly judged to be low risk across studies, with only study 1 rated as high risk in this domain. Bias due to missing outcome data (D3) was largely rated as low risk across studies, indicating minimal attrition and appropriate handling of incomplete data. Only isolated studies raised some concerns in this domain. Bias in measurement of the outcome (D4) was generally judged to be low risk, particularly in trials employing objective biochemical, physiological, or performance-related outcomes, although several studies raised some concerns. Bias in the selection of the reported results (D5) is a further important source of potential bias. While many studies were judged to raise some concerns due to limited availability of pre-specified protocols or trial registrations, a high risk of bias was identified in studies 1, 10 and 13.

Consequently, the overall risk of bias was assessed as low for a substantial proportion of trials, moderate for the majority, and high for three studies), primarily driven by factors relating to randomisation procedures and selective reporting.

### 3.3. Synthesis of Safety Outcomes

After individual study characterisation, safety data were synthesised across biomarker categories and adverse event profiles to provide an integrated assessment of SARE tolerability.

#### 3.3.1. Hepatic Function

Hepatic biomarkers, including ALT, AST, ALP, and bilirubin, were reported in nine studies involving 1578 participants. Across these studies, liver enzymes and bilirubin were reported as stable or non-significant, with only small magnitude changes where noted. For example, Naik (2024) [35] reported AST increases of 4.1% and ALT decreases of 2.3%, Mutha et al. (2025b) [39] reported minimal increases in ALT, AST, and ALP of 0.38 IU per litre, and Vani et al. (2026) [44] reported no changes in ALT, AST, ALP, or bilirubin. Pakhale et al. (2026) [46] (*n* = 1002), reported no clinically significant changes in AST or ALT after 8 weeks of 600 mg/day supplementation, with between-group differences remaining within normal laboratory reference ranges. Overall, hepatic markers remained within normal or unchanged ranges as reported.

#### 3.3.2. Renal Function

Seven studies assessed renal biomarkers, including serum creatinine and BUN in 1420 participants. Reported outcomes indicated stable renal indices or small, non-significant changes. For example, Mutha et al. (2025b) [39] reported a decrease in creatinine of 0.07 mg/dL, Naik (2024) [35] reported decreases in BUN of 1.4% and creatinine of 5.2%, and Vani et al. (2026) [44] reported no changes in creatinine or BUN. Pakhale et al. (2026) [46] similarly reported no clinically significant changes in serum creatinine following 8 weeks of 600 mg/day supplementation in 1002 adults with stress and anxiety.

#### 3.3.3. Thyroid Function

Thyroid biomarkers, including TSH, T3, and T4, were reported in two studies involving 160 participants. Both studies reported no changes in TSH, T3, or T4, indicating stable thyroid function during SARE supplementation.

#### 3.3.4. Haematological Parameters

Nine studies reported haematological parameters in 1553 participants. Measures included CBC indices and specific cell counts such as haemoglobin, haematocrit, platelets, leukocytes, and differential counts. Across these studies, haematological markers were generally reported as stable or unchanged. Where changes were noted, they were small and not described as clinically meaningful. For example, Naik (2024) [35] reported increases in haemoglobin and haematocrit of 2.3%. Pakhale et al. (2026) [46] reported no clinically significant changes in WBC, RBC, haemoglobin, haematocrit, or platelet counts after 8 weeks of 600 mg/day supplementation in 1002 participants. No study reported clinically significant haematological abnormalities attributable to SARE.

#### 3.3.5. Hormonal Biomarkers

Eight studies measured serum or salivary cortisol in 508 participants. Most studies reported reductions in cortisol, with decreases ranging from approximately 8.6% to 32.6% where quantified, including Chandrasekhar et al. (2012) [24], Gopukumar et al. (2021) [29], Salve et al. (2019) [27], and Naik (2024) [35]. One study reported no change in cortisol, as observed by Vaidya et al. (2025) [34], and one study reported mixed, sex-specific changes in salivary cortisol, as reported by Coope et al. (2026) [43].

Regarding testosterone, seven studies assessed this biomarker in 383 participants. Increases were reported in several trials, including increases of 15.3% and 29.0% in Wankhede et al. (2015) [25] and Puttaswamy et al. (2025) [41], respectively, as well as salivary increases reported by Coope et al. (2026) [43]. One study reported no change in testosterone, as observed by Mutha et al. (2025a) [40].

Five studies assessed additional hormones, including LH, FSH, DHEA-S, prolactin, estradiol, and progesterone, in 328 participants. Outcomes were generally stable or showed small shifts without reported clinical significance. For example, Mutha et al. (2025a) [40] reported no changes, Vani et al. (2026) [44] reported increases in estradiol and progesterone alongside decreases in LH and FSH, and Coope et al. (2026) [43] reported minor decreases in DHEA-S in both sexes.

#### 3.3.6. Adverse Events Summary

Mild adverse events occurred in both SARE and placebo groups and most commonly included gastrointestinal symptoms such as nausea, dyspepsia, and loose stools, as well as headache, drowsiness, and mild upper respiratory complaints. Reporting of adverse event frequency was inconsistent across studies, precluding quantitative pooling. In the largest trial to date, Pakhale et al. (2026) [46] reported 28 adverse events in 26 of 498 participants (5.2%) receiving 600 mg/day SARE compared with 46 events in 39 of 504 participants (7.8%) receiving placebo over 8 weeks, with the most commonly reported events being nausea and headache. The between-group difference was not statistically significant (χ^2^ = 1.362, *p* = 0.850), and the relative risk was 0.67 (95% CI 0.42–1.06). Ferreira et al. (2026) [45] reported no adverse events during an 8-day intervention in amateur handball players. Discontinuations due to adverse events were infrequent, and when reported, symptoms were mild and resolved upon cessation of supplementation. No clear dose–response relationship was evident for adverse events, as studies using higher doses of 600 mg per day did not report higher adverse event rates compared to lower doses of 125 to 300 mg per day. Additionally, adverse event rates did not increase with longer intervention durations, suggesting no cumulative toxicity within the studied timeframe of up to 180 days.

#### 3.3.7. Summary

In 23 randomised controlled trials encompassing 2317 participants, SARE appears to be well tolerated, characterised as follows:

- Maintenance of hepatic, renal, thyroid, and haematological biomarkers within normal clinical ranges.
- No serious adverse events attributable to supplementation.
- Mild adverse events occurred at rates comparable to placebo.
- No evidence of dose-dependent or duration-dependent toxicity within the studied parameters.

These findings provide evidence for the potential short-to-medium term safety of SARE in healthy adult populations at doses up to 600 mg/day for durations of up to 180 days.

### 3.4. GRADE

The certainty of evidence across outcomes ranges from moderate to low. While most included studies were randomised controlled trials (22/23), several methodological and contextual considerations influenced the overall confidence in findings across different outcome domains. Risk of bias was downgraded due to some methodological limitations identified using the Cochrane RoB 2 tool, with most studies rated as having some concerns and three studies rated as high risk of bias, primarily related to reporting of randomisation procedures and outcome selection.

Indirectness was considered due to the inclusion of populations described as “healthy” but encompassing diverse groups such as individuals with chronic stress, insomnia, frailty, menopausal status, or sexual dysfunction. While reflective of real-world supplement use, this may limit direct generalisability to strictly healthy populations.

Imprecision was identified due to heterogeneous sample sizes (*n* = 14–1002 per study), variable intervention durations, and the absence of pooled quantitative estimates due to heterogeneity in study design and outcome reporting. Notably, the large multi-centre trial by Pakhale et al. (2026) [46] contributed substantially greater precision for safety outcomes.

Inconsistency was generally low for objective biomarker outcomes, with hepatic, renal, thyroid, and haematological parameters showing consistent findings across studies, remaining within clinical reference ranges. Some variability was observed in hormonal outcomes, including cortisol and testosterone, reflecting differences in populations, measurement methods, and study designs.

Publication bias could not be excluded. Most studies were conducted within a single geographic region (India, *n* = 19), and a substantial proportion of records were identified via Google Scholar. In addition, the potential for industry involvement in supplement research should be considered, although this was not formally declared or assessed.

Overall, evidence for core safety biomarkers (hepatic, renal, thyroid, and haematological outcomes) was rated as moderate certainty, reflecting consistent findings across multiple randomised trials using objective clinical measures, despite some limitations in study quality and precision. Evidence for hormonal outcomes was rated as low certainty due to variability in measurement approaches. Evidence for adverse events was rated as low certainty due to variability and limited detail in reporting across studies.

## 4. Discussion

### 4.1. Biomarkers

This systematic review synthesised evidence from 23 clinical trials assessing a broad range of biochemical, hormonal, haematological, and physiological biomarkers following SARE supplementation in healthy adult populations. Across studies, hepatic and renal safety biomarkers were the most frequently assessed and showed a high degree of consistency. Key liver enzymes (ALT, AST and ALP), bilirubin and renal function indices (serum creatinine, urea and BUN) remained within established reference ranges following SARE supplementation, irrespective of dose (125–600 mg/day), formulation or duration (single dose to 180 days). Importantly, no hepatotoxic or nephrotoxic signals were observed in short-term trials, and also in the longest study included, which administered 400 mg/day for 180 days. In the context of this review, no study reported clinically relevant elevations or serious adverse trends in hepatic or renal biomarkers attributable to SARE, echoing a previous systematic review on Ashwagandha root extract (‘standardised’ not included) in human ailments [49]. The consistent maintenance of these markers within normal ranges across all included studies, including those with 180-day durations, did not demonstrate risk under studied conditions. This should be interpreted in the context of the overall moderate risk of bias identified across studies, with several trials exhibiting methodological limitations that may affect the reliability of safety reporting.

Haematological parameters remained stable across interventions. Measures, including haemoglobin, haematocrit, red and white blood cell counts, platelet counts, and differential leukocyte profiles, showed no adverse changes following SARE supplementation. This stability was observed across diverse populations, including athletes undergoing structured training, elderly individuals with frailty, and menopausal women. The absence of haematological disturbances suggests SARE showed no evidence of adverse effects on immune or haemostatic function response in healthy adults. This stability may indicate that the physiological effects attributed to SARE are unlikely to be mediated through alterations in oxygen-carrying capacity or immune cell proliferation, but rather through modulation of stress, hormonal, and inflammatory pathways [50].

Decreases in serum or salivary cortisol levels have been reported in multiple trials involving healthy individuals, athletes during intensive training phases, and the general adult population. Although not universal across all studies, these reductions were most consistently observed in trials using doses of 300–600 mg/day for 6–12 weeks. Cortisol modulation appeared to reflect modulation rather than suppression, with values remaining within physiological ranges for healthy adults, consistent with typical morning concentrations (~13 μg/dL at 06:00 and ~8 μg/dL at 09:00) and the expected diurnal decline following peak secretion [51]. This may suggest an adaptogenic effect rather than endocrine disruption [52]. Additionally, research suggests that the constituents of Ashwagandha root extract influence central stress-related pathways, including modulation of cholinergic activity, attenuation of glucocorticoid responses, and suppression of excess nitric oxide signalling within the brain [53]. In addition, the presence of antioxidant compounds may contribute to neuroprotective effects by limiting oxidative stress [54].

Several studies have demonstrated increases in serum testosterone in healthy men, physically active individuals, and mixed-sex cohorts, while remaining within normal physiological limits. In women, particularly menopausal and sexually active populations, SARE supplementation was associated with modest improvements in estradiol, progesterone and gonadotropin balance without exceeding reference ranges. No study has reported hormonal abnormalities suggestive of endocrine dysregulation with SARE. Thyroid levels remained within normal limits and were not accompanied by changes in free T4 or T3, suggesting physiological variation rather than pathological thyroid stimulation or suppression. The clinical relevance of these hormonal changes remains unclear, particularly in long-term use or in populations with endocrine sensitivity. This finding contrasts with previous research on case reports documenting thyroid injury following Ashwagandha [55,56]. In the 12-month clinical trial of SARE on safety, no thyroid changes were observed across 191 participants [16]. This divergence between controlled trial evidence and isolated case reports highlights a discrepancy between clinical trial findings and post-marketing observations, which may reflect differences in extract standardisation, formulation, exposure duration, or individual susceptibility. It also suggests that rare or idiosyncratic adverse effects may not be captured within the relatively short duration and controlled conditions of clinical trials, showing the need for further mechanistic investigation and pharmacovigilance data. There is also the matter of potential heavy metal contamination in various adaptogenic herbal supplements [57] associated with noncancerous thyroid disease [58]; however, this is beyond the scope of this systematic review.

### 4.2. Adverse Event Reporting

The most commonly reported adverse events were non-specific and low severity, including variations in gastrointestinal discomfort (nausea, abdominal pain, diarrhoea), headache, drowsiness and mild upper respiratory symptoms. These events were typically isolated or did not require medical intervention. Symptoms such as these are commonly reported in intervention trials and are not unexpected in studies involving oral supplementation [59].

The absence of serious adverse events across diverse populations, including healthy adults, athletes exposed to high training loads, elderly individuals with frailty and menopausal women, strengthens the generalisability of the safety findings. Mild adverse events were minimal within the controlled settings of the included trials. However, the discussion of adverse events must also acknowledge limitations in reporting quality. Several studies reported “no adverse events” without clearly specifying monitoring methods. Others did not quantify adverse event frequency or severity using standardised criteria. This heterogeneity limits the precision with which adverse event incidence can be compared across trials and raises the possibility of under-reporting, particularly in open-label or non-randomised designs [60]. Therefore, the absence of reported adverse events should not be interpreted as definitive evidence of zero risk, but rather as an indication of no observed clinically relevant harm within the constraints of the study designs. Consequently, the absence of reported adverse events should be interpreted with caution and may not reflect the true incidence of rare or delayed effects.

### 4.3. Regulatory Considerations and Toxicological Limitations

The adverse event profile of SARE appears generally comparable with that of other widely consumed botanical supplements. For example, green tea extract, another popular supplement, has been associated with idiosyncratic hepatotoxicity in rare cases, with some instances requiring liver transplantation [61,62]. St. John’s Wort carries significant drug interaction risks due to potent CYP3A4 and P-glycoprotein induction, necessitating careful monitoring when co-administered with medications [63]. Ginkgo biloba has been associated with increased bleeding risk, particularly when combined with anticoagulants [64].

In contrast, SARE’s adverse events were predominantly mild, transient, and occurred at rates that were statistically indistinguishable from those in the placebo group. No serious adverse events, hospitalisations, or life-threatening reactions attributable to SARE were documented across 2317 participants. The maintenance of clinical biomarkers within normal ranges suggests a low risk of significant harm when SARE is consumed according to the study protocols (125–600 mg/day for up to 180 days) by healthy adults. These findings suggest that the safety profile of Ashwagandha is linked to the use of standardised extracts and medium-length intervention durations in healthy participants. The contrast between the consistent safety outcomes reported in clinical trials and the occasional adverse effects described in real-world use highlights the importance of factors, such as duration of supplementation, product quality, and individual sensitivity [65,66,67,68].

Preclinical and mechanistic toxicology studies were not systematically evaluated in this review, even though a body of animal research examining toxicity, dose response relationships, and point-of-departure thresholds does exist and underpins the overall safety assessment of Ashwagandha. Nevertheless, this review prioritises human data to ensure direct applicability to healthy participants. Such data are critical for the establishment of health-based guidance values, safety margins, and the identification of organ-specific toxicities [69]. Although the identified trials report mild to no adverse events, there is little data to assess idiosyncratic or delayed adverse reactions because the studies are sub chronic (6–12 weeks) in exposure. Recent regulatory concerns across the EU have underscored the requirement for dedicated and targeted toxicological assessments that extend beyond the scope of this review [70,71]. 

As in many human studies, conventional clinical chemistry panels are used as surrogates for adverse event monitoring. In the case of SARE, it is well established in preclinical studies that high doses are hepatotoxic [72], as in some case studies [73,74], and more specific biomarkers, such as keratin-18 fragments, glutamate dehydrogenase, or miR-122 [75] may be useful.

Second, the review is specific to a healthy population; however, the average consumer includes vulnerable populations, such as the elderly, pregnant women, or those with pre-existing hepatic, renal, or endocrine conditions [69]. The labelling of food supplements may be a reasonable risk mitigation factor in these populations because the dose is controlled. However, in ordinary foods eaten for pleasure rather than physiological effects and not in a dose form, label warnings may not provide adequate protection.

Finally, the focus on healthy subjects, not on medication, avoids the risk of potential drug–nutrient interactions (DNIs), which are critical given that 70–80% of medications [76] are metabolised by CYP450, which can bemodulated by SARE [74]. This issue is further complicated by the heterogeneity of SARE formulations, which result in varying levels of withanolides, a key active compound in possible DNIs [77]. The standardisation of withanolide content across SARE formulations may therefore be important to reduce variability and clarify their interaction potential.

Future toxicological research should prioritise dose-escalation studies, chronic toxicity assessments, reproductive and developmental toxicity evaluations [78], and mechanistic investigations into reported cases of hepatotoxicity to provide more robust evidence base for regulatory decision-making and clinical recommendations [69,73]. The primary aim of future trials should be to conduct dedicated chronic dosing studies at preclinical and Phase I levels to address regulatory concerns. Such studies should be sufficiently powered to establish any cumulative toxicity and/or delayed adverse events [32].

### 4.4. Limitations

Several limitations of this systematic review should be acknowledged. The available evidence remains relatively limited, particularly with respect to long-term safety outcomes. The review included a restricted number of databases and was limited to articles published in English, which may have resulted in the omission of relevant studies. The predominance of a single formulation (KSM-66) across included trials may limit generalisability, as safety outcomes may not fully represent other SARE supplements with differing extraction processes or withanolide profiles.

Methodological heterogeneity across the included studies represents an additional limitation. Considerable variability was observed in study design (randomised controlled trials, open-label designs), SARE formulations (1.5–5% withanolides; ~3.3-fold variation), dosages (125–600 mg/day; 4.8-fold variation), and intervention durations (single dose to 180 days) and outcome measures, limiting direct comparisons between trials and precluding quantitative synthesis. Although many studies reported favourable safety profiles, several trials were characterised by small sample sizes, short intervention periods, open-label designs, or incomplete reporting of randomisation and allocation procedures, contributing to concerns identified in the risk of bias assessment. Most studies recruited young or middle-aged participants, with comparatively few trials involving older adults or exclusively female cohorts. Most studies were conducted in India (*n* = 19), with limited representation from Western populations. This geographic concentration may limit generalisability because of potential differences in genetic polymorphisms (CYP450 variants), baseline health status, dietary backgrounds and phytochemical exposure. One of the included studies was authored by a member of the review team, which may represent a potential source of bias, although study selection, data extraction, and synthesis followed predefined eligibility criteria. Additionally, although all included studies reported no external funding, the reporting of funding sources and potential conflicts of interest was not detailed beyond these statements.

This heterogeneity precluded a formal meta-analysis and quantitative pooling of biomarker data. Although qualitative synthesis was conducted systematically, the inability to generate pooled effect estimates limits the precision of safety characterisation. Future studies would benefit from greater standardisation in dosing, biomarker protocols, and outcome reporting.

## 5. Conclusions

This systematic review synthesised evidence from 23 clinical trials (*n* = 2317 participants) evaluating the safety and tolerability of SARE in generally healthy adults. Across studies, no clinically meaningful adverse changes in hepatic, renal, haematological, or endocrine biomarkers were observed, and no serious adverse events attributable to SARE were reported. Mild adverse events were infrequent, non-specific, and comparable to placebo. Doses of 125–600 mg/day for up to 180 days were well tolerated, with no evidence of dose-dependent or cumulative toxicity within the studied durations. Interpretation is limited by the short duration of most trials, heterogeneous sample sizes and populations, and variability in adverse event reporting, with potential reporting and publication bias. Accordingly, while SARE appears well-tolerated under controlled conditions, further large-scale, long-term studies are needed to confirm safety. Future research should prioritise extended-duration trials (>12 months) and standardisation of extract composition. Regulatory frameworks would benefit from harmonised safety thresholds and standardised specifications for Ashwagandha extracts, including total withanolide content and composition. 

## Figures and Tables

**Table 1:** Database-specific search strategies and keyword combinations.

Databases	Search Strategy
PubMed	(*Withania somnifera* [Title/Abstract] OR Ashwagandha [Title/Abstract]) AND (root extract OR standardised OR standardised extract) AND (safety OR tolerability OR adverse events OR side effects OR toxicity OR biomarkers) AND (clinical trial OR randomised controlled trial)
Web of Science	(TI = (Ashwagandha OR *Withania somnifera*) OR AB = (Ashwagandha OR *Withania somnifera*)) AND (TI = (root extract OR standardised OR standardised extract) OR AB = (root extract OR standardised OR standardised extract)) AND (TI = (safety OR tolerability OR adverse events OR toxicity OR biomarkers) OR AB = (safety OR tolerability OR adverse events OR toxicity OR biomarkers))
Google Scholar	Ashwagandha standardised root extract, healthy, safety, tolerability, and adverse events biomarkers

**Table 2:** Characteristics of included randomised and controlled trials investigating SARE.

Study	Study Design	Country	Participants (*n*)	Dose (mg/Day)	Duration
Alluri et al., (2021) [28]	Open-label, pharmacokinetic randomised crossover	India	Healthy male volunteers (*n* = 14)	600	Single dose
Chandrasekhar et al. (2012) [24]	RCT	India	Healthy adults with chronic stress (*n* = 64)	600	60 days
Chauhan et al. (2022) [33]	RCT	India	Healthy adult males (*n* = 50)	600	8 weeks
Choudhary et al. (2015) [26]	RCT	India	Healthy athletic adults (*n* = 50)	600	12 weeks
Coope et al. (2026) [43]	RCT	Spain	Healthy adult athletes (*n* = 56)	600	6 weeks
Ferreira et al. (2026) [45]	RCT	Portugal	Healthy male amateur handball players (*n* = 31)	600	8 days
Gopukumar et al. (2021) [29]	RCT	India	Healthy stressed adults (*n* = 130)	300	90 days
Jówko et al. (2025) [42]	RCT	Poland	Healthy male non-athletes (*n* = 33)	600	8 weeks
Langade et al. (2021) [30]	RCT	India	Healthy adults and insomnia patients (*n* = 80)	600	8 weeks
Mahadevan et al. (2025) [38]	RCT	India	Healthy stressed adults (*n* = 90)	125	12 weeks
Mutha et al. (2025a) [40]	RCT	India	Healthy women with female sexual dysfunction (*n* = 62)	600	8 weeks
Mutha et al. (2025b)—Male [39]	RCT	India	Healthy men (*n* = 100)	600	8 weeks
Naik (2024) [35]	RCT	India	Elderly with frailty (*n* = 50)	600	8 weeks
Pakhale et al. (2026) [46]	RCT	Multi-centre	Adults with stress and anxiety (*n* = 1002)	600	8 weeks
Puttaswamy et al. (2025) [41]	RCT	India	Healthy males (*n* = 40)	600	6 weeks
Raut (2024) [36]	RCT	India	Healthy volunteers (*n* = 60)	500	60 days
Salve et al. (2019) [27]	RCT	India	Healthy stressed adults (*n* = 60)	250–600	8 weeks
Tiwari et al. (2021) [31]	RCT	India	Healthy athletic adults (*n* = 50)	600	8 weeks
Vaidya et al. (2025) [34]	RCT	India	Healthy male volunteers (*n* = 18)	400	180 days
Vani et al. (2026) [44]	RCT	India	Menopausal women (*n* = 60)	600	8 weeks
Verma et al. (2021) [32]	RCT	India	Healthy adults (*n* = 80)	600	8 weeks
Verma et al. (2024) [37]	RCT	India	Healthy, physically active adults (*n* = 80)	600	8 weeks
Wankhede et al. (2015) [25]	RCT	India	Healthy males (*n* = 57)	600	8 weeks

**Table 3:** Summary of intervention protocols, clinical safety biomarkers and adverse events reported in studies assessing SARE supplementation in healthy adults.

Author and Year	Duration	Intervention	Objective	Biomarkers	Adverse Events	Outcomes
Alluri et al. (2021) [28]	16 days	SARE (sustained-release; Prolanza™), 300 mg, 5% withanolides (15 mg/capsule), single dose (2 capsules), 600 mg, single dose Comparator: SARE (KSM-66), 300 mg, 5% withanolides (15 mg/capsule), single dose (2 capsules), 600 mg, single dose	To assess the comparative pharmacokinetics and safety of SARE sustained-release capsules compared to a reference formulation in healthy adults under fasting conditions	AST ALT Total bilirubin Serum creatinine Urea	None described	↔ AST ↔ ALT ↔ Bilirubin ↔ Creatinine ↔ Urea
Chandrasekhar et al. (2012) [24]	60 days	SARE (KSM-66), 300 mg, ≥5% withanolides, 2× daily, 600 mg/day, 60 days	To evaluate the safety and efficacy of a high-concentration full-spectrum extract of SARE in reducing stress and anxiety and improving the general well-being of adults who were under stress	Serum cortisol Serum globulin Serum triglycerides	SARE: 6 mild AEs (nasal congestion [*n* = 1], constipation [*n* = 1], URI symptoms [*n* = 1], drowsiness [*n* = 1], decreased appetite [*n* = 1], cough/cold [*n* = 1]) Placebo: 5 mild AEs (dry mouth [*n* = 1], fatigue [*n* = 1], fever [*n* = 1], headache [*n* = 1], abdominal pain/diarrhoea [*n* = 1]) SAEs: none reported	↓ Cortisol −27.9% vs. baseline; placebo −7.9% ↔ Globulin ↔ Triglycerides
Chauhan et al. (2022) [33]	56 days	SARE (KSM-66), 300 mg, ≥5% withanolides, 2× daily, 600 mg/day, 56 days	To evaluate the effect of SARE on improving sexual health in adult males	Serum testosterone Serum prolactin	SARE: 4 mild AEs (sleepiness [*n* = 2], mild abdominal pain [*n* = 1], low-grade joint pain [*n* = 1]) Placebo: 3 mild AEs (abdominal pain [*n* = 1], mild diarrhoea [*n* = 2]) SAEs: none reported	↑ Testosterone +17.9% vs. baseline; placebo +1.3% ↓ Prolactin −3.9% vs. baseline; placebo +8.3%
Choudhary et al. (2015) [26]	84 days	SARE (KSM-66), 300 mg, 5% withanolides, 2× daily, 600 mg/day, 84 days	To evaluate the efficacy of SARE in enhancing cardiorespiratory endurance and improving QOL in healthy athletic adults	VO_2_ max HR Systolic BP Diastolic BP Respiratory rate	None described	↑ VO_2_ max +13.6% vs. baseline; placebo +4.4% ↔ HR ↔ Systolic BP ↔ Diastolic BP ↔ Respiratory rate
Coope et al. (2026) [43]	42 days	SARE (KSM-66), 600 mg, 5% withanolides, 1× daily, 600 mg/day, 42 days	To investigate the effects of 600 mg/day SARE on physiological stress biomarkers, perception of recovery, muscle strength and aerobic capacity in team sports athletes during pre-season training	Salivary cortisol Salivary cortisone Salivary testosterone Salivary DHEA-S Salivary alpha-amylase	SARE: 1 mild AE (headache [*n* = 1]) Placebo: 1 mild AE (gastrointestinal discomfort [*n* = 1]) SAEs: none reported	↓ Salivary cortisone −0.9% females ↓ Salivary cortisone −30.7% males ↓ Salivary cortisol −18.3% males ↑ Salivary cortisol +5.3% females ↑ Salivary testosterone +22.3% females ↑ Salivary testosterone +12.4% males ↓ Salivary DHEA-S −28.7% females ↓ Salivary DHEA-S −11.3% males ↑ Salivary alpha-amylase +45.0% females ↓ Salivary alpha-amylase −0.6% males
Ferreira et al. (2026) [45]	8 days	SARE 600 mg, 1× daily, 600 mg/day, 8 days	To evaluate the effects of 8 days of 600 mg/day Ashwagandha supplementation on neuromuscular performance and recovery in amateur male handball players	Handgrip strength Knee extension strength Knee flexion strength Perceived muscle soreness Rating of fatigue TQR	No AEs reported during the 8-day intervention SAEs: none reported	↑ Handgrip strength ↔ Knee extension strength ↔ Knee flexion strength ↔ Perceived soreness, fatigue and recovery
Gopukumar et al. (2021) [29]	90 days	SARE (sustained-release; Prolanza™), 300 mg, 5% withanolides (15 mg/capsule), 1× daily, 300 mg/day, 90 days	To evaluate the efficacy and safety of SARE sustained-release capsule on cognitive functions, stress levels, sleep quality, overall well-being and safety in healthy adults experiencing stress	Serum cortisol BDNF CBC AST ALT Serum creatinine	None described	↓ Serum cortisol −29.9% vs. baseline ↔ BDNF ↔ CBC ↔ AST ↔ ALT ↔ Serum creatinine
Jówko et al. (2025) [42]	56 days	SARE (KSM-66), 300 mg, 5% withanolides, 2× daily, 600 mg/day, 56 days	To evaluate the effects of SARE (600 mg/day) on aerobic capacity, muscle oxygenation and resting blood haematological parameters in healthy male non-athletes undergoing 8 weeks of HIIT	VO_2_ max Haemoglobin Haematocrit RBC count Leukocyte count Lymphocyte count Monocyte count Total granulocyte count Eosinophil count Basophil count Platelet count Blood lactate concentration HR SmO_2_	None described	↑ VO_2_ max +3.3% vs. baseline ↔ Haemoglobin ↔ Haematocrit ↔ RBC count ↔ Leukocyte count ↔ Lymphocyte count ↔ Monocyte count ↔ Total granulocyte count ↔ Eosinophil count ↔ Basophil count ↔ Platelet count ↔ Blood lactate concentration ↔ HR ↔ SmO_2_
Langade et al. (2021) [30]	56 days	SARE (KSM-66), 300 mg, 5% withanolides, 2× daily, 600 mg/day, 56 days	To evaluate the pharmacological effect of SARE on sleep in healthy subjects and those with insomnia, and to assess its efficacy and safety compared to a placebo for insomnia and anxiety	HR BP	None described	↔ HR ↔ BP
Mahadevan et al. (2025) [38]	84 days	SARE (Zenroot™), 125 mg, 1.5% withanolides, 1× daily, 125 mg/day, 84 days	To evaluate the safety and efficacy of SARE formulation Zenroot in reducing stress, anxiety, mood disturbances and sleep issues in individuals with non-chronic mild to moderate stress	Serum cortisol Salivary alpha-amylase	SARE: mild AEs in 33.3% of participants (pyrexia [*n* = 6, mild], cough/cold [*n* = 1, moderate]) Placebo: AEs in 20% of participants (pyrexia [*n* = 4, including 1 moderate]) SAEs: none reported	↓ Serum cortisol −16.8% vs. baseline ↔ Salivary alpha-amylase
Mutha et al. (2025a) [40]	56 days	SARE (KSM-66), 300 mg, 5% withanolides, 2× daily, 600 mg/day, 56 days	To compare the efficacy and safety of SARE against a placebo for improving female sexual dysfunction in healthy women	E2 Serum progesterone FSH LH Serum prolactin Serum testosterone Serum creatinine Serum bilirubin ALT AST	SARE: no AEs reported Placebo: 1 mild AE (dizziness [*n* = 1]) SAEs: none reported	↔ E2 (NS) ↔ Progesterone (NS) ↔ FSH (NS) ↔ LH (NS) ↔ Prolactin (NS) ↔ Testosterone (NS) ↔ Creatinine (NS) ↔ Bilirubin (NS) ↔ ALT (NS) ↔ AST (NS)
Mutha et al. (2025b) [39]	56 days	SARE (KSM-66), 300 mg, 5% withanolides, 2× daily, 600 mg/day, 56 days	To evaluate the efficacy and safety of SARE on improving sexual health in healthy adult men	Semen volume Sperm concentration Total sperm count Total sperm motility Semen pH Sperm vitality Normal sperm morphology Serum testosterone DHT FSH LH Serum prolactin Haemoglobin Serum creatinine BUN Serum bilirubin Serum albumin Serum globulin ALT AST ALP	None described	↑ Semen volume +25.56% ↑ Sperm concentration +47.72% ↑ Total sperm count +47.72% ↔ Total sperm motility ↔ Semen pH ↑ Sperm vitality +18.70% ↑ Normal sperm morphology +4.33 percentage points ↑ Testosterone +70.46 ng/dL ↑ DHT +37.64 pg/mL ↓ FSH −0.19 IU/L ↑ LH +0.29 IU/L ↑ Prolactin +0.70 ng/mL ↔ Haemoglobin ↓ Creatinine −0.07 mg/dL ↑ BUN +0.37 IU/L (NS) ↓ Bilirubin −0.07 mg/dL ↓ Albumin −0.14 g/dL ↓ Globulin −0.08 g/dL ↑ ALT +0.38 IU/L ↑ AST +0.38 IU/L ↑ ALP +0.38 IU/L
Naik et al. (2024) [35]	56 days	SARE (KSM-66), 300 mg, 5% withanolides, 2× daily, 600 mg/day, 56 days	To evaluate the efficacy and safety of SARE in improving frailty and quality of life in elderly individuals	CRP Cortisol CK Haemoglobin Haematocrit Serum bilirubin Total cholesterol ALP BUN Serum creatinine AST ALT	SARE: 5 mild AEs total (headache, nausea, vomiting, body pain [*n* = 5 total]) Placebo: 1 mild AE (stomach pain [*n* = 1]) SAEs: none reported	↓ CRP −2.5% ↓ Cortisol −8.6% ↓ CK −1.5% ↑ Haemoglobin +2.3% ↑ Haematocrit +2.3% ↑ Bilirubin +0.6% ↓ Total cholesterol −2.3% ↑ ALP +8.7% ↓ BUN −1.4% ↓ Serum creatinine −5.2% ↑ AST +4.1% ↓ ALT −2.3%
Pakhale et al. (2026) [46]	8 weeks	SARE (KSM-66), 5% withanolides, 300 mg 2× daily, 600 mg/day, 8 weeks	To evaluate the safety and tolerability of 8-week administration of 600 mg/day Ashwagandha root extract in adults with self-reported stress and anxiety in a large multi-centre trial	WBC count RBC count Haematocrit Haemoglobin Platelet count AST ALT Creatinine GATT Adverse event incidence	SARE: 28 events in 26 participants (5.2% of *n* = 498); most common nausea (2.0%), dry mouth (1.4%), diarrhoea (0.6%), vomiting (0.4%), headache (0.2%) Placebo: 46 events in 39 participants (7.8% of *n* = 504); most common nausea (3.2%), headache (2.2%), dry mouth (1.4%), diarrhoea (0.4%), drowsiness (0.4%) Between-group difference: not statistically significant (χ^2^ = 1.362, *p* = 0.850) SAEs: none reported Deaths or withdrawals due to AEs: none	↔ WBC ↔ RBC ↔ Haematocrit ↔ Haemoglobin ↔ Platelets ↔ AST ↔ ALT ↔ Creatinine ↔ Tolerability (88.2% vs. 89.1%) ↓ AEs (RR 0.67)
Puttaswamy et al. (2025) [41]	42 days	SARE (ASVAMAN^®^), 300 mg, 2.5% withanolides, 2× daily, 600 mg/day, 42 days	To evaluate the effects of SARE (ASVAMAN^®^) on the energy and endurance in healthy adults	Serum cortisol Serum testosterone	SARE: no AEs reported (1 dropout; reason not stated) Placebo: no AEs reported (1 dropout; reason not stated) SAEs: none reported	↓ Serum cortisol −14.5% ↑ Serum testosterone +29.0%
Raut et al. (2024) [36]	60 days	SARE (Agewel™), 250 mg, 1.5% withanolides, 2× daily, 500 mg/day, 60 days	To evaluate the effects of SARE on biomarkers of inflammation and muscle status in response to exercise and to assess its safety in healthy volunteers	hs-CRP IL-6 TNF-α Serum myostatin VO_2_ max	None described	↓ hs-CRP −27.8% ↓ IL-6 −23.6% ↓ TNF-α −19.4% ↓ Myostatin −14.5% ↑ VO_2_ max +5.4%
Salve et al. (2019) [27]	56 days	SARE (KSM-66), 125–300 mg, 5% withanolides, 2× daily, 250–600 mg/day, 56 days	To evaluate the effect of an aqueous SARE in reducing stress and anxiety in adults and to assess the dose–response relationship of high-concentration root extract on sleep quality, psychometric stress scales and serum cortisol levels	Serum cortisol	None described	↓ Cortisol −16.5% (250 mg) ↓ Cortisol −32.6% (600 mg)
Tiwari et al. (2021) [31]	56 days	SARE (KSM-66), 300 mg, >5% withanolides, 2× daily, 600 mg/day, 56 days	To evaluate the efficacy and safety of SARE in enhancing cardiorespiratory endurance in healthy athletic adults	VO_2_ max Antioxidant levels	SARE: 1 mild AE (ear pain [*n* = 1]) Placebo: 3 mild AEs (diarrhoea [*n* = 2], very low-grade fever [*n* = 1]) SAEs: none reported	↑ VO_2_ max +15.4% ↑ Antioxidant levels +12.3%
Vaidya et al. (2025) [34]	180 days	SARE (LongeFera), 200 mg, ≥2.5% withanolides, 2× daily, 400 mg/day, 180 days	To investigate the safety of SARE capsules in healthy adult male and female participants, and to establish safety and tolerability by recording adverse events	CBC Fasting blood glucose CD3 CD4 CD8 CRP Cortisol Testosterone NT-proBNP	None described	↔ CBC ↔ Fasting blood glucose ↑ CD3 +4.2% ↑ CD4 +6.1% ↑ CD8 +3.8% ↓ CRP −51.1% ↔ Cortisol ↑ Testosterone +15.7% ↓ NT-proBNP −28.7%
Vani et al. (2026) [44]	56 days	SARE (KSM-66), 300 mg, 5% withanolides, 2× daily, 600 mg/day, 56 days	To assess the efficacy and safety of SARE for managing menopausal symptoms in women	E2 Progesterone LH FSH Serum creatinine BUN ALT AST ALP Bilirubin Haemoglobin RBC count Haematocrit Total leukocyte count Lymphocytes Monocytes Eosinophils Basophils Absolute neutrophil count Platelet count	SARE: 1 mild AE (cough/cold [*n* = 1]) Placebo: 2 mild AEs (stomach-ache [*n* = 1], indigestion [*n* = 1]) SAEs: none reported	↑ E2 +11.6% ↑ Progesterone +9.4% ↓ LH −14.7% ↓ FSH −18.5% ↔ Creatinine ↔ BUN ↔ ALT ↔ AST ↔ ALP ↔ Bilirubin ↔ Haemoglobin ↔ RBC count ↔ Haematocrit ↔ Total leukocyte count ↔ Lymphocytes ↔ Monocytes ↔ Eosinophils ↔ Basophils ↔ Absolute neutrophil count ↔ Platelet count
Verma et al. (2021) [32]	56 days	SARE (KSM-66), 300 mg, 5% withanolides, 2× daily, 600 mg/day, 56 days	To evaluate the safety of SARE consumption in healthy adults	Haemoglobin Neutrophil count Platelet count ALP AST ALT TSH T3 T4	None described	↔ Haemoglobin ↔ Neutrophil count ↔ Platelet count ↔ ALP ↔ AST ↔ ALT ↔ TSH ↔ T3 ↔ T4
Verma et al. (2024) [37]	56 days	SARE (KSM-66), 300 mg, 5% withanolides, 2× daily, 600 mg/day, 56 days	To investigate the effects of SARE on muscle size, strength and cardiorespiratory endurance in healthy adults performing resistance training and to assess its safety and tolerability	Haemoglobin Neutrophils Platelet count ALP AST ALT T3 T4 TSH Body temperature Pulse rate Respiratory rate Systolic BP Diastolic BP	None described	↔ Haemoglobin ↔ Neutrophils ↔ Platelet count ↔ ALP ↔ AST ↔ ALT ↔ T3 ↔ T4 ↔ TSH ↔ Body temperature ↔ Pulse rate ↔ Respiratory rate ↔ Systolic BP ↔ Diastolic BP
Wankhede et al. (2015) [25]	56 days	SARE (KSM-66), 300 mg, 5% withanolides, 2× daily, 600 mg/day, 56 days	To examine the possible effects of SARE consumption on muscle mass and strength in healthy young men engaged in resistance training	Serum CK Serum testosterone	None described	↓ Serum CK −27.9% ↑ Serum testosterone +15.3%

Abbreviations: SARE, standardised Ashwagandha root extract; KSM-66, proprietary root-only Ashwagandha extract standardised to ≥5% withanolides; AE, adverse event; SAE, serious adverse event; URI, upper respiratory infection; NS, non-significant; RR, relative risk; ↑, increased; ↓, decreased; ↔, unchanged. Biomarkers: AST, aspartate aminotransferase; ALT, alanine aminotransferase; ALP, alkaline phosphatase; BUN, blood urea nitrogen; CRP, C-reactive protein; hs-CRP, high-sensitivity C-reactive protein; CK, creatine kinase; BDNF, brain-derived neurotrophic factor; CBC, complete blood count; WBC, white blood cell; RBC, red blood cell; BP, blood pressure; HR, heart rate; E2, estradiol; DHEA-S, dehydroepiandrosterone sulfate; DHT, dihydrotestosterone; FSH, follicle-stimulating hormone; LH, luteinising hormone; TSH, thyroid-stimulating hormone; T3, triiodothyronine; T4, thyroxine; NT-proBNP, N-terminal pro–B-type natriuretic peptide; IL-6, interleukin-6; TNF-α, tumour necrosis factor-alpha; CD3, CD4, CD8, T-cell surface differentiation markers; VO_2_ max, maximal oxygen consumption; SmO_2_, muscle oxygen saturation; TQR, Total Quality Recovery; GATT, Global Assessment of Tolerability to Therapy; QOL, quality of life; HIIT, high-intensity interval training.

## Data Availability

No new data were created or analysed in this study. Data sharing is not applicable to this article.

## Supplementary Materials

The following supporting information can be downloaded at: https://www.mdpi.com/article/10.3390/ph19050725/s1, File S1: PRISMA 2020 Checklist.
