# Supplementary material for: Back to the Roots: Safety and Tolerability of Standardised Ashwagandha (Withania somnifera) Root Extract in Healthy Adults—A Systematic Review of Biomarkers and Adverse Events
Source: Pharmaceuticals (Basel). 2026 May 2;19(5):725. doi: 10.3390/ph19050725 (PMC13209861; doi:10.3390/ph19050725)
Supplement: Supplementary file 1 [file pharmaceuticals-19-00725-s001.zip › pharmaceuticals-4262404-supplementary/PRISMA_2020_checklist_FILLED.pdf]

PRISMA 2020 Checklist

Section and Topic	Item #	Checklist item	Location where item is reported
**TITLE**			
Title	1	Identify the report as a systematic review.	Title (page 1) — the report is explicitly identified as a 'Systematic Review' in the title.
**ABSTRACT**			
Abstract	2	See the PRISMA 2020 for Abstracts checklist.	Abstract (page 1), structured under Background, Methods, Results and Conclusions in line with the PRISMA 2020 for Abstracts checklist.
**INTRODUCTION**			
Rationale	3	Describe the rationale for the review in the context of existing knowledge.	Section 1 (Introduction), paragraphs 1–4, pages 2–3. Rationale situates SARE within the broader growth of the supplement market, existing case reports of hepatotoxici

PRISMA

PRISMA 2020 Checklist

Section and Topic	Item #	Checklist item	Location where item is reported
			ty, and gaps regarding standardised root-only formulations.
Objectives	4	Provide an explicit statement of the objective(s) or question(s) the review addresses.	Section 1 (Introduction), final paragraph, page 3. Explicit aim: to critically evaluate and synthesise evidence on the safety and tolerability of standardised Ashwagandha root extract (SARE) in healthy adult populations, with a focus on clinical biomarkers and adverse event reporting.
**METHODS**			
Eligibility criteria	5	Specify the inclusion and exclusion criteria for the review and how studies were grouped for the syntheses.	Section 2.1 (Materials and Methods — PICOS), pages 3–4. Inclusion

PRISMA 2020 Checklist

Section and Topic	Item #	Checklist item	Location where item is reported
			and exclusion criteria are defined using the PICOS framework (Population, Intervention, Comparator, Outcomes, Study design). Grouping for synthesis is defined in Section 2.4 (Outcomes) and Section 3.4 (Synthesis of Safety Outcomes).
Information sources	6	Specify all databases, registers, websites, organisations, reference lists and other sources searched or consulted to identify studies. Specify the date when each source was last searched or consulted.	Section 2.2 (Search Keywords), page 4. Databases: PubMed, Web of Science and Google Scholar. Studies published from 2010 to April 2026 were considered. No registers, websites, or reference-list

PRISMA 2020 Checklist

Section and Topic	Item #	Checklist item	Location where item is reported
			searches were performed; Scopus was excluded due to indexing overlap.
Search strategy	7	Present the full search strategies for all databases, registers and websites, including any filters and limits used.	Section 2.2 (Search Keywords) and Table 1 (Database-specific search strategies and keyword combinations), page 4. Full Boolean search strings are provided for each database.
Selection process	8	Specify the methods used to decide whether a study met the inclusion criteria of the review, including how many reviewers screened each record and each report retrieved, whether they worked independently, and if applicable, details of automation tools used in the process.	Section 2.3 (Data Extraction) and Section 2.5 (Study Selection), pages 4–5. Two reviewers (O.C.C. and A.L.) screened records independently using a piloted, standardised extraction

PRISMA

PRISMA 2020 Checklist

Section and Topic	Item #	Checklist item	Location where item is reported
			form; discrepancies were resolved by discussion. No automation tools were used.
Data collection process	9	Specify the methods used to collect data from reports, including how many reviewers collected data from each report, whether they worked independently, any processes for obtaining or confirming data from study investigators, and if applicable, details of automation tools used in the process.	Section 2.3 (Data Extraction), page 4. Data were extracted independently by two reviewers (O.C.C. and A.L.) using a standardised, piloted extraction form (Supplementary Material 3). Discrepancies were resolved through discussion; corresponding authors were contacted when data were missing/unclear (two-week response window). No automation

PRISMA 2020 Checklist

Section and Topic	Item #	Checklist item	Location where item is reported
			tools were used.
Data items	10a	List and define all outcomes for which data were sought. Specify whether all results that were compatible with each outcome domain in each study were sought (e.g. for all measures, time points, analyses), and if not, the methods used to decide which results to collect.	Section 2.4 (Outcomes) , page 5. Primary outcomes: clinical safety biomarkers (liver enzymes, renal function, haematological indices, inflammatory and hormonal markers) and adverse events (frequency, type, severity). Clinically significant thresholds were pre-defined using CTCAE criteria (Hy's Law, creatinine changes, TSH/free T4, haematological reference ranges). Secondary outcomes (physiologic

PRISMA 2020 Checklist

Section and Topic	Item #	Checklist item	Location where item is reported
			al, cognitive, performance) were described where relevant.
	10b	List and define all other variables for which data were sought (e.g. participant and intervention characteristics, funding sources). Describe any assumptions made about any missing or unclear information.	Section 2.3 (Data Extraction), page 4. Extracted variables included author/year, country, study design, participant characteristics, sample size, intervention and comparator details, withanolide content, manufacturer, duration, outcomes assessed, adverse events and main findings. Assumptions about missing data are stated (authors were contacted when data were

PRISMA 2020 Checklist

Section and Topic	Item #	Checklist item	Location where item is reported
			unclear).
Study risk of bias assessment	11	Specify the methods used to assess risk of bias in the included studies, including details of the tool(s) used, how many reviewers assessed each study and whether they worked independently, and if applicable, details of automation tools used in the process.	Section 3.3 (Risk of Bias Assessment) and Figure 3, pages 13–14. Risk of bias was assessed using the Cochrane Risk of Bias 2 (RoB 2) tool across five domains (D1–D5). Independence of assessors/automation tools is not explicitly stated in the paper.
Effect measures	12	Specify for each outcome the effect measure(s) (e.g. risk ratio, mean difference) used in the synthesis or presentation of results.	Section 2.7 (Statistical Analysis), page 5. No meta-analysis was conducted; results were presented as reported in the primary studies (between-group differences, percentage changes,

PRISMA 2020 Checklist

Section and Topic	Item #	Checklist item	Location where item is reported
			and the individual study statistical outputs, typically $p < 0.05$).
Synthesis methods	13a	Describe the processes used to decide which studies were eligible for each synthesis (e.g. tabulating the study intervention characteristics and comparing against the planned groups for each synthesis (item #5)).	Section 2.1 (PICOS) and Section 2.7 (Statistical Analysis), pages 3–5. Studies eligible for each synthesis category were selected according to the a priori PICOS criteria and grouped by biomarker category and by adverse events.
	13b	Describe any methods required to prepare the data for presentation or synthesis, such as handling of missing summary statistics, or data conversions.	Section 2.7 (Statistical Analysis), page 5. Data were summarised descriptively without re-analysis. Percentage changes and between-group

PRISMA 2020 Checklist

Section and Topic	Item #	Checklist item	Location where item is reported
			differences were extracted as reported; no data conversion or imputation was performed.
	13c	Describe any methods used to tabulate or visually display results of individual studies and syntheses.	Section 2.7 (Statistical Analysis), page 5; Tables 2 and 3 and Figures 1–3 (study characteristics, biomarker findings, PRISMA flow diagram, geographical distribution, and RoB 2 summary).
	13d	Describe any methods used to synthesize results and provide a rationale for the choice(s). If meta-analysis was performed, describe the model(s), method(s) to identify the presence and extent of statistical heterogeneity, and software package(s) used.	Section 2.7 (Statistical Analysis), page 5. A meta-analysis was not conducted owing to heterogeneity in study design, intervention protocols and

PRISMA 2020 Checklist

Section and Topic	Item #	Checklist item	Location where item is reported
			outcome reporting. A structured qualitative synthesis was performed, organised by biomarker category and adverse events.
	13e	Describe any methods used to explore possible causes of heterogeneity among study results (e.g. subgroup analysis, meta-regression).	Not applicable — no meta-analysis was performed, so no formal subgroup or meta-regression was conducted. Heterogeneity is discussed narratively in Section 3.5 (GRADE) and Section 4.4 (Limitations).
	13f	Describe any sensitivity analyses conducted to assess robustness of the synthesized results.	Not applicable — no quantitative synthesis was performed,

PRISMA 2020 Checklist

Section and Topic	Item #	Checklist item	Location where item is reported
			so no sensitivity analyses were conducted.
Reporting bias assessment	14	Describe any methods used to assess risk of bias due to missing results in a synthesis (arising from reporting biases).	Section 2.6 (Certainty of Evidence) and Section 3.5 (GRADE), pages 5 and 18. Publication bias was considered within the GRADE framework (one of the five downgradin g domains).
Certainty assessment	15	Describe any methods used to assess certainty (or confidence) in the body of evidence for an outcome.	Section 2.6 (Certainty of Evidence), page 5. The GRADE framework was used; evidence was initially rated as high and downgrade d based on risk of bias, inconsisten cy, indirectness , imprecision, and publication bias, with

PRISMA 2020 Checklist

Section and Topic	Item #	Checklist item	Location where item is reported
			final ratings of high, moderate, low or very low.
**RESULTS**			
Study selection	16a	Describe the results of the search and selection process, from the number of records identified in the search to the number of studies included in the review, ideally using a flow diagram.	Section 2.5 (Study Selection), page 5 and Figure 1 (PRISMA 2020 flow diagram), page 6. 702 records identified (PubMed n=51, Google Scholar n=618, Web of Science n=33); 145 duplicates removed; 557 screened; 514 excluded at title/abstract; 43 full-texts assessed; 22 excluded with reasons; 23 studies included.
	16b	Cite studies that might appear to meet the inclusion criteria, but which were excluded, and explain why they were excluded.	Section 2.5 (Study Selection), page 5. Reasons for exclusion at

PRISMA 2020 Checklist

Section and Topic	Item #	Checklist item	Location where item is reported
			full-text stage: leaf rather than root extract (n=4), additional active ingredients (n=2), lack of standardisation (n=1), non-healthy participants (n=3), non-randomised trials (n=3), and absence of biomarker measurements from saliva/urine/serum (n=9). Individual excluded studies are not cited by name.
Study characteristics	17	Cite each included study and present its characteristics.	Section 3.1 (Study Characteristics) and Table 2 (Characteristics of included randomised and controlled trials investigating SARE), pages 6–8. Full details

PRISMA 2020 Checklist

Section and Topic	Item #	Checklist item	Location where item is reported
			of the 23 included studies are provided, and geographical distribution is shown in Figure 2.
Risk of bias in studies	18	Present assessments of risk of bias for each included study.	Section 3.3 (Risk of Bias Assessment) and Figure 3 (RoB 2 traffic-light/summary plot), pages 13–14. Per-study judgments for all five RoB 2 domains and overall judgment are presented.
Results of individual studies	19	For all outcomes, present, for each study: (a) summary statistics for each group (where appropriate) and (b) an effect estimate and its precision (e.g. confidence/credible interval), ideally using structured tables or plots.	Section 3.1 (Study Characteristics) and Table 3 (Summary of intervention protocols, clinical safety biomarkers and adverse events), pages 8–

PRISMA 2020 Checklist

Section and Topic	Item #	Checklist item	Location where item is reported
			13. For each study, intervention details, biomarkers measured, reported adverse events and effect estimates (percentage changes) are tabulated. Confidence intervals were not consistently reported in the primary studies.
Results of syntheses	20a	For each synthesis, briefly summarise the characteristics and risk of bias among contributing studies.	Section 3.4 (Synthesis of Safety Outcomes) and its sub-sections 3.4.1 (Hepatic), 3.4.2 (Renal), 3.4.3 (Thyroid), 3.4.4 (Haematological), 3.4.5 (Hormonal), 3.4.6 (Adverse Events) and 3.4.7 (Summary), pages 14–17. Each synthesis is

Section and Topic	Item #	Checklist item	Location where item is reported
			introduced with the number of contributing studies and participants, and risk-of-bias is referenced to Figure 3.
	20b	Present results of all statistical syntheses conducted. If meta-analysis was done, present for each the summary estimate and its precision (e.g. confidence/credible interval) and measures of statistical heterogeneity. If comparing groups, describe the direction of the effect.	Section 3.4.1–3.4.7 (Synthesis of Safety Outcomes), pages 14–17. As no meta-analysis was undertaken, results are presented as a structured qualitative summary of biomarker changes (direction of effect, magnitude where reported) and adverse event frequencies. A narrative comparison between SARE and placebo groups is provided, noting the

PRISMA 2020 Checklist

Section and Topic	Item #	Checklist item	Location where item is reported
			non-significant between-group difference ($\chi^2 = 1.362$, $p = 0.850$; RR 0.67, 95% CI 0.42–1.06) in the largest trial (Pakhale et al., 2026).
	20c	Present results of all investigations of possible causes of heterogeneity among study results.	Not applicable — no meta-analysis or formal investigation of statistical heterogeneity was conducted. Heterogeneity across populations, doses, durations and formulations is discussed qualitatively in Section 3.5 (GRADE) and Section 4.4 (Limitations).
	20d	Present results of all sensitivity analyses conducted to assess the robustness of the synthesized results.	Not applicable — no sensitivity

PRISMA 2020 Checklist

Section and Topic	Item #	Checklist item	Location where item is reported
			analyses were conducted because no quantitative synthesis was performed.
Reporting biases	21	Present assessments of risk of bias due to missing results (arising from reporting biases) for each synthesis assessed.	Section 3.5 (GRADE), page 18. Publication bias is addressed qualitatively: most studies were from a single region (India, n = 20), a substantial proportion of records came from Google Scholar, and potential industry involvement could not be fully excluded.
Certainty of evidence	22	Present assessments of certainty (or confidence) in the body of evidence for each outcome assessed.	Section 3.5 (GRADE), page 18. Certainty was rated as moderate for core biomarkers (hepatic,

Section and Topic	Item #	Checklist item	Location where item is reported
			renal, thyroid, haematological) and low for hormonal outcomes and adverse events, with explicit reasoning for each downgrade.
**DISCUSSION**			
Discussion	23a	Provide a general interpretation of the results in the context of other evidence.	Section 4.1 (Biomarkers) and Section 4.2 (Adverse Event Reporting), pages 18–21, and Section 4.3 (Regulatory Considerations and Toxicological Limitations), pages 21–22. Findings are interpreted relative to prior systematic reviews, observational safety data, case reports and comparator botanicals (e.g., green

PRISMA 2020 Checklist

Section and Topic	Item #	Checklist item	Location where item is reported
			tea, St John's Wort, ginkgo biloba).
	23b	Discuss any limitations of the evidence included in the review.	Section 3.3 (Risk of Bias) and Section 4.4 (Limitations), pages 13 and 22. Limitations include moderate risk of bias in many trials, heterogeneity in dose (125–600 mg/day), duration (single dose to 180 days) and withanolide content (1.5–5%), and scarce long-term data.
	23c	Discuss any limitations of the review processes used.	Section 4.4 (Limitations), page 22. Review-process limitations include restricted databases, English-language only, no grey

Section and Topic	Item #	Checklist item	Location where item is reported
			literature, predominance of KSM-66 and Indian studies, inclusion of a study authored by a review team member, and the decision not to conduct a meta-analysis.
	23d	Discuss implications of the results for practice, policy, and future research.	Section 4.3 (Regulatory Considerations and Toxicological Limitations) and Section 5 (Conclusion), pages 21–23. Implications are set out for practice (tolerability in healthy adults at studied doses), policy (harmonised safety thresholds and standardisation of extract composition

PRISMA

PRISMA 2020 Checklist

Section and Topic	Item #	Checklist item	Location where item is reported
			) and future research (extended-duration trials >12 months, vulnerable populations, drug–herb interactions, chronic toxicity and developmental studies).
**OTHER INFORMATION**			
Registration and protocol	24a	Provide registration information for the review, including register name and registration number, or state that the review was not registered.	Abstract, page 1; Section 1 (Introduction), final paragraph, page 3; and Section 2.1 (PICOS), page 3. The review is registered in PROSPERO under ID CRD420261337116.
	24b	Indicate where the review protocol can be accessed, or state that a protocol was not prepared.	Section 2.1 (PICOS), page 3. The protocol was registered with PROSPERO (ID CRD420261337116) and can be accessed

PRISMA 2020 Checklist

Section and Topic	Item #	Checklist item	Location where item is reported
			via the PROSPERO register. A completed PRISMA checklist is provided as Supplementary Material 1.
	24c	Describe and explain any amendments to information provided at registration or in the protocol.	No amendments to the registered PROSPERO protocol are reported in the manuscript.
Support	25	Describe sources of financial or non-financial support for the review, and the role of the funders or sponsors in the review.	Funding statement (end of manuscript). 'This research received no external funding.' No funder or sponsor had a role in the review.
Competing interests	26	Declare any competing interests of review authors.	Conflicts of Interest statement (end of manuscript). M.E.T.W., M.J.T., T.J.S. and B.R.-V. declare no conflict of

Section and Topic	Item #	Checklist item	Location where item is reported
			interest. O.C.C. and A.L. disclose consulting and speaking activities for companies in the dietary supplements industry (including some distributing the supplement investigated), undertaken independently of this research.
Availability of data, code and other materials	27	Report which of the following are publicly available and where they can be found: template data collection forms; data extracted from included studies; data used for all analyses; analytic code; any other materials used in the review.	Data Availability Statement (end of manuscript): 'No new data were created or analysed in this study.' The PRISMA checklist is provided as Supplementary Material 1 and the data extraction form as Supplementary Material

PRISMA 2020 Checklist

Section and Topic	Item #	Checklist item	Location where item is reported
			3.

From: Page MJ, McKenzie JE, Bossuyt PM, Boutron I, Hoffmann TC, Mulrow CD, et al. The PRISMA 2020 statement: an updated guideline for reporting systematic reviews. BMJ 2021;372:n71. doi: 10.1136/bmj.n71. This work is licensed under CC BY 4.0. To view a copy of this license, visit https://creativecommons.org/licenses/by/4.0/
